# Identification of disulfiram as a secretase-modulating compound with beneficial effects on Alzheimer’s disease hallmarks

**DOI:** 10.1038/s41598-018-19577-7

**Published:** 2018-01-22

**Authors:** Sven Reinhardt, Nicolai Stoye, Mathias Luderer, Falk Kiefer, Ulrich Schmitt, Klaus Lieb, Kristina Endres

**Affiliations:** 1 Department of Psychiatry and Psychotherapy, University Medical Center of the Johannes Gutenberg University, Mainz, Germany; 2 Central Institute of Mental Health (CIMH), Department of Addictive Behavior and Addiction Medicine, Medical Faculty Mannheim, University of Heidelberg, Mannheim, Germany

## Abstract

ADAM10 is a metalloproteinase acting on the amyloid precursor protein (APP) as an alpha-secretase in neurons. Its enzymatic activity results in secretion of a neuroprotective APP cleavage product (sAPP-alpha) and prevents formation of the amyloidogenic A-beta peptides, major hallmarks of Alzheimer’s disease (AD). Elevated ADAM10 levels appeared to contribute to attenuation of A-beta-plaque formation and learning and memory deficits in AD mouse models. Therefore, it has been assumed that ADAM10 might represent a valuable target in AD therapy. Here we screened a FDA-approved drug library and identified disulfiram as a novel ADAM10 gene expression enhancer. Disulfiram increased ADAM10 production as well as sAPP-alpha in SH-SY5Y human neuronal cells and additionally prevented A-beta aggregation in an *in vitro* assay in a dose-dependent fashion. In addition, acute disulfiram treatment of Alzheimer model mice induced ADAM10 expression in peripheral blood cells, reduced plaque-burden in the dentate gyrus and ameliorated behavioral deficits. Alcohol-dependent patients are subjected to disulfiram-treatment to discourage alcohol-consumption. In such patients, enhancement of ADAM10 by disulfiram-treatment was demonstrated in peripheral blood cells. Our data suggest that disulfiram could be repurposed as an ADAM10 enhancer and AD therapeutic. However, efficacy and safety has to be analyzed in Alzheimer patients in the future.

## Introduction

Alzheimer’s disease is the most prevalent dementia in the aging population. With up to 15 million cases worldwide it represents a disease with high impact on medicinal care burden and patient numbers are estimated to increase further in the following years (e.g.^1^). The fact that the majority of diagnosed Alzheimer cases represent the sporadic, non-genetic form of the disease emphasizes why successful treatment has remained elusive. The origin of the sporadic form still remains enigmatic, hampering a targeted therapy in humans (reviewed in^2^). One of the pivotal hallmarks of the neurodegenerative process is the production, oligomerization, deposition, and degradation of the Amyloid-beta (A-beta) peptide (see^3^). This has led to numerous therapeutic approaches, targeting single steps of A-beta metabolism which so far failed (for an overview on recent clinical trials^4^). An alternative to such mono-functional drugs might be offered by drugs that interfere with several pathways associated with the disease, therefore acting as multifunctional drugs (for example^5–7^).

Two proteases compete for cleavage of APP: ADAM10 (A disintegrin and metalloproteinase 10^8^) and BACE-1 (beta site APP cleaving enzyme-1^9^). Shifting the balance toward the alpha-secretase ADAM10 not only prevents generation of neurotoxic A-beta peptides but simultaneously increases levels of the soluble APP fragment sAPPs-alpha *in vivo*^10–12^. The latter has been shown to be neuroprotective and to enhance bouton density in the brains of intracranially infused mice^13^. Elevating ADAM10 resulted in increased learning and memory of AD model mice and strongly diminished plaque deposition^10^, while a reduction in BACE-1 levels also rescued AD phenotypes^14,15^. Manipulating the interplay between both of these proteases has been suggested as an attractive potential target in therapy development^16,17^. If such a drug could also interfere in a beneficial manner with other pathways contributing to AD – such as hyperphosphorylation of the cytoskeleton-associated protein Tau by glycogen synthase kinase 3 beta (GSK3beta)^18^ – this might be of high interest. To identify such novel drug candidates for AD treatment, we performed an initial screening of more than 600 FDA-approved drugs to identify candidates regulating transcriptional activity of both genes. We identified disulfiram as one of the most promising candidates and evaluated it further in cells and in mice, as well as in an observatory clinical study. In this study advantage was taken of the fact that alcohol-dependent patients are often given disulfiram to discourage the consumption of alcohol. The so-called disulfiram-ethanol reaction is due to increased serum acetaldehyde concentrations resulting from lack of clearance via aldehyde dehydrogenase, one of the enzymes that are blocked by disulfiram. The discomfort associated with this syndrome (nausea, sweating, chest pain etc.) is intended to serve as a discouraging stimulus. The use of disulfiram in this clinical indication facilitated measurements of ADAM10 expression within peripheral blood cells in humans before and after two weeks of treatment with the newly identified drug.

## Results

### Identification of disulfiram as an alpha-secretase enhancer from a FDA-approved drug library

**Figure 1:**
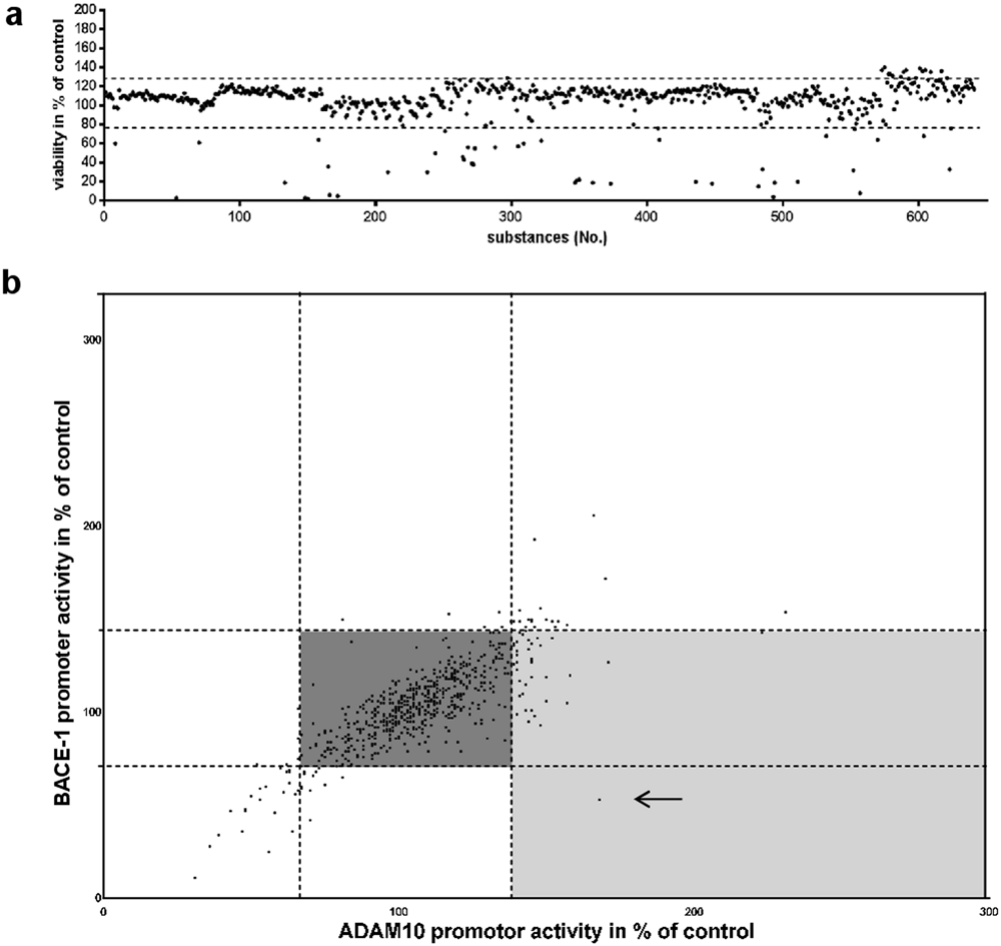
Screening of a FDA-approved drug library for inducers of ADAM10 promoter activity enhancers. (**a**) SH-SY5Y cells were incubated for 48 h with 0.1% v/v substance and viability assessed using the Cell Titer Glo assay. DMSO (solvent) served as control and values obtained for solvent-treated cells were set to 100%. Dashed lines indicate the maximum tolerated proliferative or toxic effect. Concentrations were subsequently adjusted by further dilution until no toxic/proliferative effect could be observed. 13 substances were excluded from further analyses due to their remaining high toxic potential (for final conc. see Suppl. Table 1). (**b**) SH-SY5Y cells were transiently transfected with a dual reporter vector for both promoter activities, human ADAM10 and BACE-1 (20). Subsequently, cells were incubated with the respective drug or DMSO as a solvent-control (values set to 100%). The experiment was conducted three-times independently (SDs are not shown for clarity of the graph). The main part of the tested drugs remained without effects (dark grey box) while a small number of drugs revealed promoter- inducing potential (light grey box). The most promising candidate, i.e. high ADAM10 promoter activity and low BACE-1 promoter activity (disulfiram), is indicated by an arrow.

In previous studies, identification of novel ADAM10 enhancers from phytomedical collections via reporter gene assay has been demonstrated to be a promising approach^19,20^. By analyzing drugs already approved by the FDA, rapid translation into clinical studies is likely, enhancing the attractiveness of such re-purposing strategies. Here, a library of 640 FDA-approved drugs was tested for potential influence on ADAM10 and BACE-1 transcriptional activity in neuronal cells. A non-toxic dosage for human, neuronal SH-SY5Y cells was assessed by starting from a 1: 3064 dilution with subsequent dilutions for compounds that resulted in >120 or <80% of viability after 48 h of incubation (see Fig. 1a for the initial results, table with final concentrations: Suppl. Table 1). The initial dilution was chosen to reach a final concentration of 2 µM for acitretin, which served as an internal ADAM10-inducing control^21^. 627 out of 640 tested substances were found to be applicable for further testing.

SH-SY5Y cells were subsequently transiently transfected with a dual reporter vector for both promoter activities, human ADAM10 and BACE-1 and incubated for 24 h with the respective drugs. From the tested library, 42 compounds were potential drug candidates as they either decreased the BACE-1 promoter activity or increased ADAM10 promoter activity so that a ratio for ADAM10/BACE1 promoter activity of minimally 1.25 was reached. For acitretin which has been already demonstrated to be a valuable ADAM10 enhancer^21^ an ADAM10 promoter activity of 142% was obtained while BACE1 promoter activity remained unchanged (110%, ratio: 1.29, Fig. 1b). One of the most interesting candidates identified by the screen was disulfiram, which not only increased ADAM10 promoter activity to 168% of control but also decreased BACE1 promoter activity to 53% of control (ratio: 3.17). We further investigated the potential of disulfiram as a novel AD drug candidate.

### Disulfiram shifts APP processing toward the non-amyloidogenic pathway in neuronal cells

**Figure 2:**
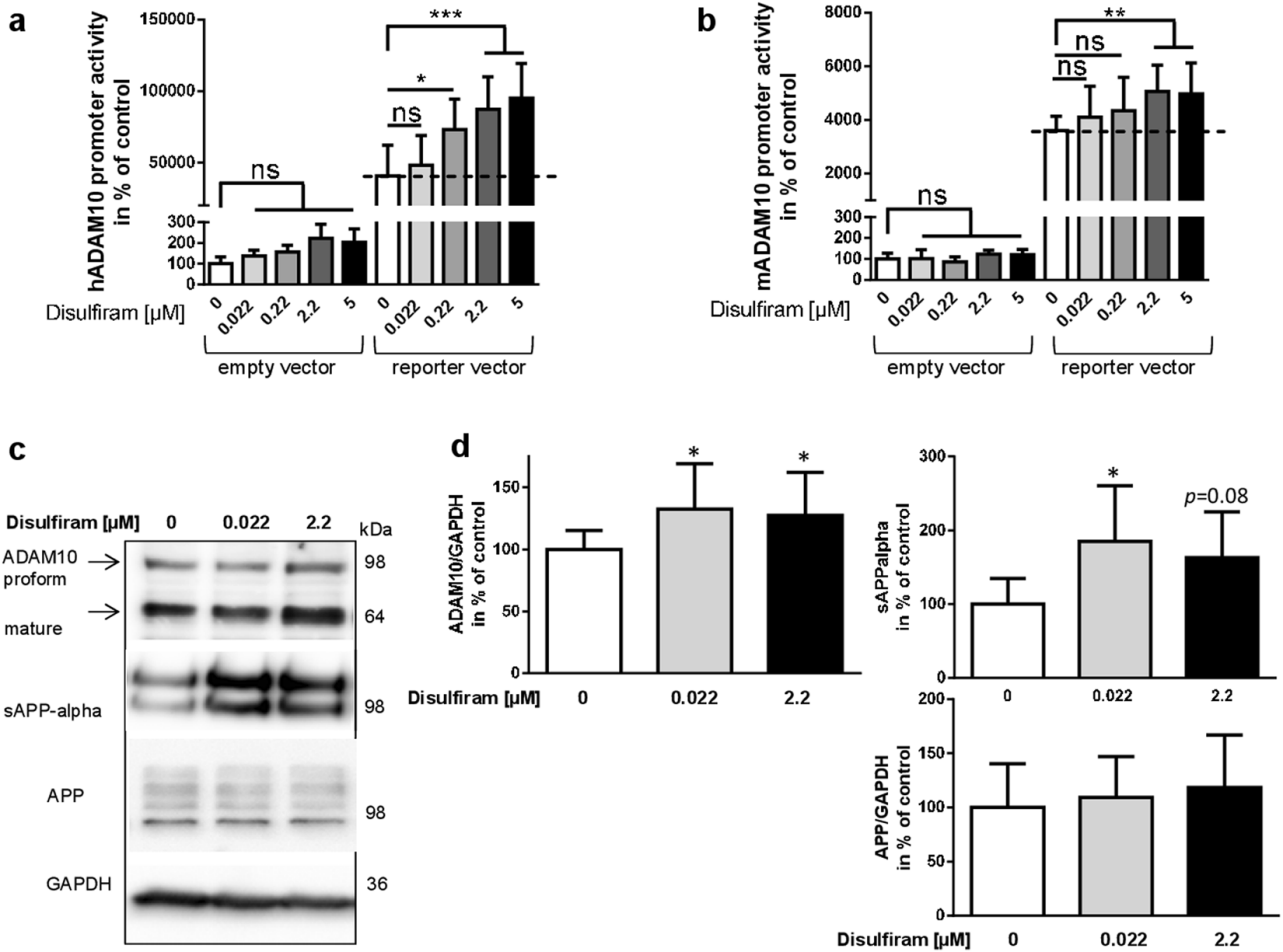
Disulfiram increases ADAM10 transcription dose-dependently and leads to enhanced ADAM10 protein amount and activity. SH-SY5Y cells were transiently transfected with a reporter vector for human ADAM10 (hADAM10) (**a**) murine ADAM10 (mADAM10) (**b**) or the empty vector as a negative control. Subsequently, cells were incubated with disulfiram in the indicated concentration or DMSO as a solvent-control (values set to 100%). The experiment was conducted three-times independently in duplicates, values were normalized to protein content of the cell lysate and are represented as mean + SD. Statistical analysis: One Way ANOVA with Tukey’s multiple comparisons test; ***p < 0.001; **p < 0.01; *p < 0.05; ns, p > 0.05). (**c**) For protein analysis, SH-SY5Y cells were incubated for 48 h with disulfiram as indicated or DMSO as solvent-control. Cell lysate adjusted for protein content was used for quantitation of ADAM10, full-length APP (antibody against APP C-terminus) and GAPDH; culture supernatants were analyzed in regard to sAPP-alpha (antibody 6E10, directed against the N-terminus). Representative blots are shown. Blots were cropped for combining the figure, single blot parts are divided by a white space. For full-length blot pictures please see Suppl. Figure 1. (**d**) Values obtained by densitometrical analysis for the specific membrane-tethered proteins were normalized to values obtained for GAPDH for quantitative analyses. Experiments were performed at least three-times independently in duplicate (n ≥ 6). Statistical analysis: One Way ANOVA *p < 0.05).

Disulfiram was tested in the screening at a concentration of 2.2 µM. Further analysis indicated that concentrations higher than 5 µM induced cytotoxicity (data not shown). Therefore, drug concentration in the cell culture experiments was restricted to a maximum of 5 µM. We first demonstrated dose-dependency of disulfiram-evoked increase in ADAM10 transcriptional activity by treating SH-SY5Y cells transfected with an ADAM10 promoter-reporter vector with different concentrations of disulfiram (Fig. 2a). While the empty control vector did not respond to disulfiram in any tested concentration, disulfiram increased ADAM10 promoter activity gaining significance for 0.22, 2.2 and 5 µM. This dose-dependent increase was also observed for the murine Adam10 promoter reporter although the results were only significant for concentrations of 2.2 µM and 5 µM (Fig. 2b).

**Figure 3:**
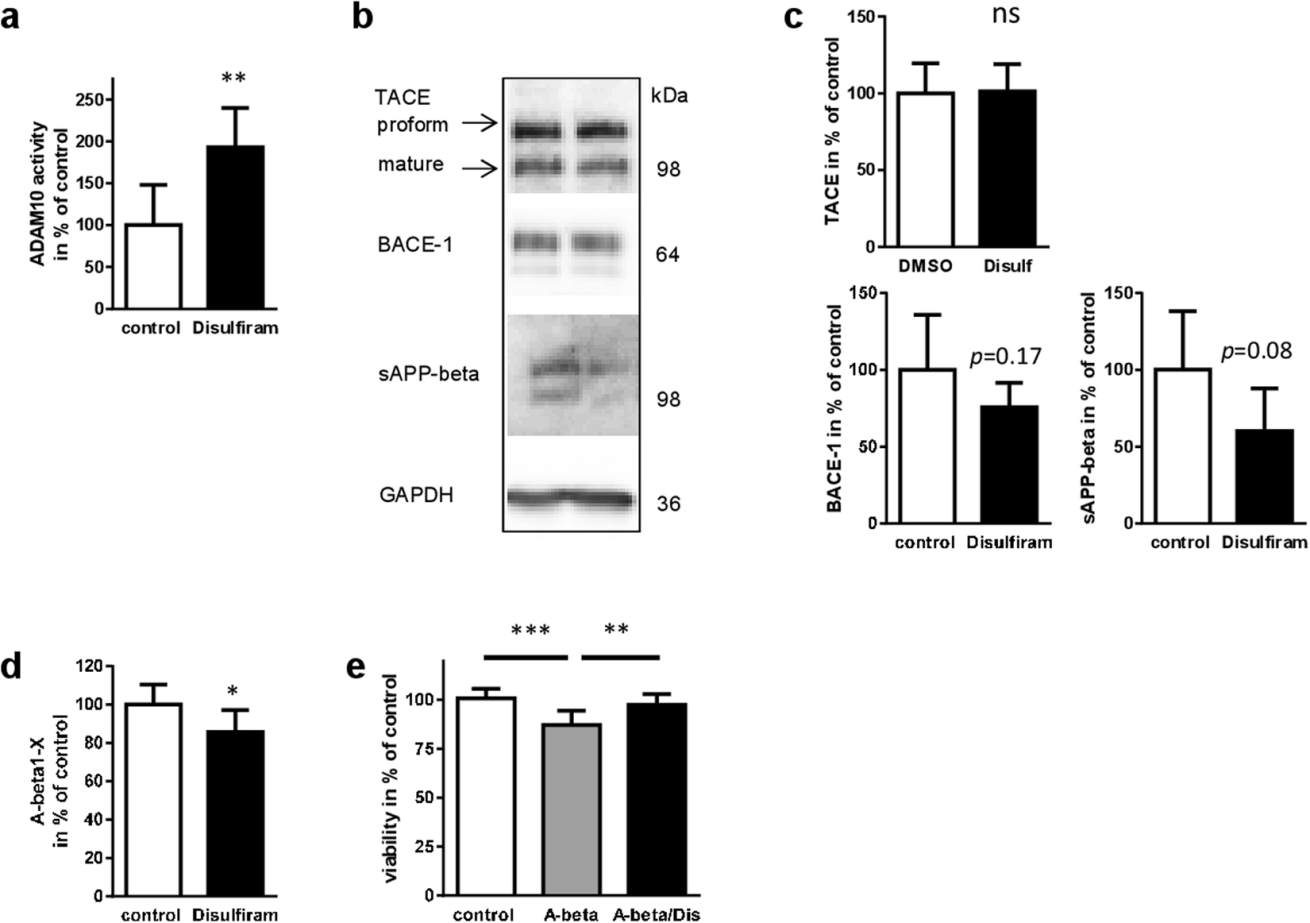
Disulfiram selectively increases ADAM10 expression and leads to decrease in A-beta production. SH-SY5Y cells were incubated with disulfiram at a concentration of 2.2 µM, or DMSO as a solvent-control (values set to 100%) for 48 h. (**a**) The cell lysate was used for measurement of ADAM10 activity by using a pro-fluorescent specific substrate. Additionally, aliquots of cell lysate were subjected to SDS-PAGE and Western blotting for analysis of levels of TACE and BACE-1. GAPDH served as a loading control. Soluble proteins from cell supernatants after 5 h of secretion were precipitated by trichloroacetic acid and subjected to SDS-PAGE and Western blotting with a sAPP-beta specific antibody (**b**). All measured densitometric values were normalized to values obtained for GAPDH of the respective sample (**c**) (n ≥ 5 for all quantifications). For TACE, the sum of mature and proform was quantified. Cropped blot pictures are separated by a white space, for full-length blot pictures please see Suppl. Figure 2. A-beta peptide secretion (A-beta 1-x) was measured by ELISA in samples from a 16 h secretion period (**d**). Statistical analysis: unpaired Student’s t-test (***p < 0.001; *p < 0.05; ns, p > 0.05). (**e**) Cells treated for 48 h with solvent (control), human A-beta_42_ peptides or peptides in combination with disulfiram (A-beta/Dis) were analyzed for viability (n = 15, two independent experiments). Statistical analysis: One Way ANOVA **p < 0.01; ***p < 0.001).

Effects observed in promoter reporter assays can yield false positives due to the fact that an isolated DNA sequence is analyzed in the absence of the physiological environment and proper chromosomal location. Therefore, the impact of disulfiram on endogenous ADAM10 protein levels in SH-SY5Y cells was analyzed. Already with the lower concentration of 0.022 µM, ADAM10 protein amount was significantly elevated (Fig. 2c and d, the sum of proform and mature form is presented). This also led to increased amounts of sAPP-alpha (185% of control) while APP full-length protein was not affected. The finding of elevated ADAM10 activity was also confirmed by measuring directly enzymatic activity in cell lysates of SH-SY5Y cells treated for 48 h with disulfiram using a pro-fluorescent peptide substrate (Fig. 3a). ADAM10 activity in drug-treated cells nearly was two-fold higher than activity in solvent-treated cells. In addition to ADAM10, ADAM17 (also designated as tumor necrosis factor alpha cleaving enzyme, TACE) can act as an alpha-secretase and lead to elevated sAPP-alpha levels (Endres *et al*., 2003). Moreover, peptide subtsrates in commercial enzyme activity kits do not always show perfect specificity. The protein level of TACE was thus analyzed after disulfiram-treatment: no difference could be observed in solvent- and in drug-treated SH-SY5Y cells (Fig. 3b,c; p = 0.9). For the beta-secretase BACE-1 a down-regulation was observed in the initial dual promoter assay and could also be confirmed in a single promoter reporter vector for human BACE-1 (data not shown). Analysis of BACE-1 protein and its cleavage product sAPP-beta revealed reduction which was not statistically significant (Fig. 3b,c). Nonetheless, a reduction of A-beta peptides of about 20% as compared to solvent-treated cells was achieved by 48 h treatment with disulfiram (Fig. 3d). Neuronal cells treated with synthetic A-beta_42_ peptides exhibited a diminished viability as shown in Fig. 3e. Co-treatment with disulfiram over a time period of 48 h was able to reverse this toxic effect and such cells were indistinguishable from control-treated cells (p = 0.65).

**Figure 4:**
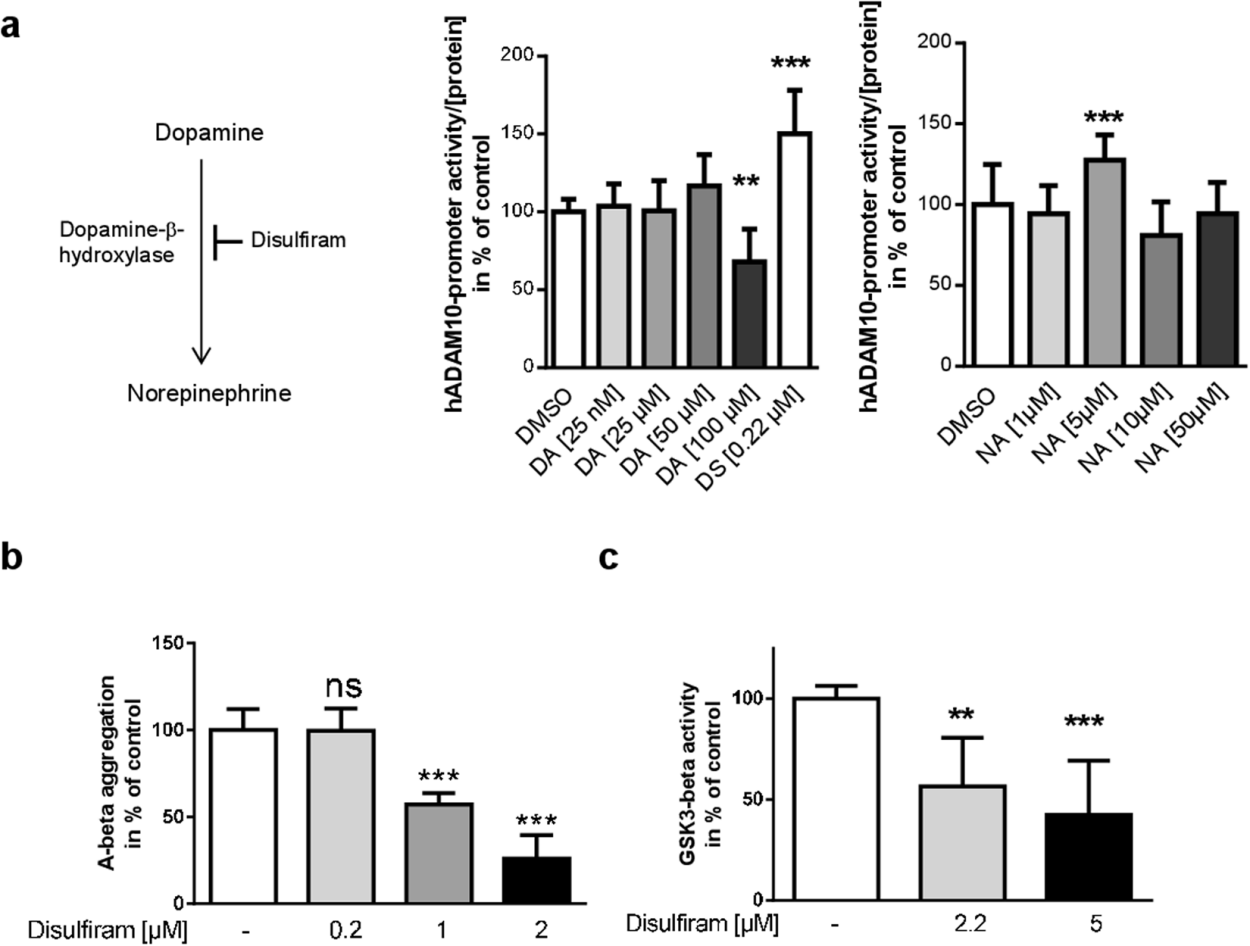
Disulfiram does not act via dopamine-metabolism on ADAM10 gene expression but has additional beneficial side effects in regard to Alzheimer’s disease. (**a**) SH-SY5Y cells were transiently transfected with a reporter vector for human ADAM10 (hADAM10) and incubated with 0.22 µM disulfiram or dopamine or norepinephrine in the indicated concentration. DMSO served as a solvent-control (values set to 100%). The experiment was conducted three-times independently in duplicates, values were normalized to protein content of the cell lysate and are represented as mean + SD. Statistical analysis: One Way ANOVA with Dunnett’s multiple comparisons test; ***p < 0.001; **p < 0.01. (**b**) To investigate a potential effect of disulfiram on A-beta aggregation, the fluorescent Thioflavin T test and human A-beta_42_ peptides were used. Peptides were supplemented with disulfiram as indicated or with the solvent at 37 °C. Three independent experiments were conducted (n ≥ 5) and slopes of fluorescence increase used for quantitation. The slopes obtained for the control were set to 100%. Statistical analysis was performed by One Way ANOVA with Bonferroni’s multiple comparisons test (***p < 0.001). (**c**) GSK3beta activity was measured by a commercial *in vitro* kit in two independent experiments performed in duplicates. Measurement values for control-treated reactions were set to 100% and values are presented as mean + SD. Statistical analysis was performed by One Way ANOVA with Dunnett’s multiple comparisons test (***p < 0.001; **p < 0.01).

We next adressed potential mechanisms of disulfiram-mediated increase of ADAM10. Interestingly, ADAM10 can be activated by dopaminergic agonists^22^. As disulfiram is a known inhibitor of dopamine-beta-hydroxylase, this might explain the increase in ADAM10 expression. Dopamine would be expected to accumulate with disulfiram-treatment while norepinephrine levels should decrease (Fig. 4a). SH-SY5Y cells are a suitable model to investigate this hypothesis, as these cells have been described to exhibit functional dopamine metabolism (reviewed in^23^). However, when SH-SY5Y cells were incubated with increasing amounts of dopamine the activity of the human ADAM10 promoter was reduced by about 40% as compared to control (100 µM dopamine, Fig. 4b). Incubation with norepinephrine resulted in a statistical significant increase of reporter gene activity to 125% of control for 5 µM while all other tested concentrations remained without effect. This result strongly suggests the effect of disulfiram on ADAM10 gene expression is independent of dopamine metabolism.

In a further assessment of the effects of disulfiram on aspects of AD pathogenesis, we found that disulfiram inhibited the aggregation of A-beta *in vitro* with 1 and 2 µM (Fig. 4b). Moreover, with 2.2 and 5 µM disulfiram an inhibition of Tau-kinase GSK3beta was seen *in vitro* (Fig. 4c). This in sum indicates that disulfiram might have the potential for being a multifunctional therapeutic compound with both, potential to prevent amyloidogenic aggregation as well as over-activation of Tau kinase.

### ADAM10-enhancing properties of disulfiram in AD model mice

**Figure 5:**
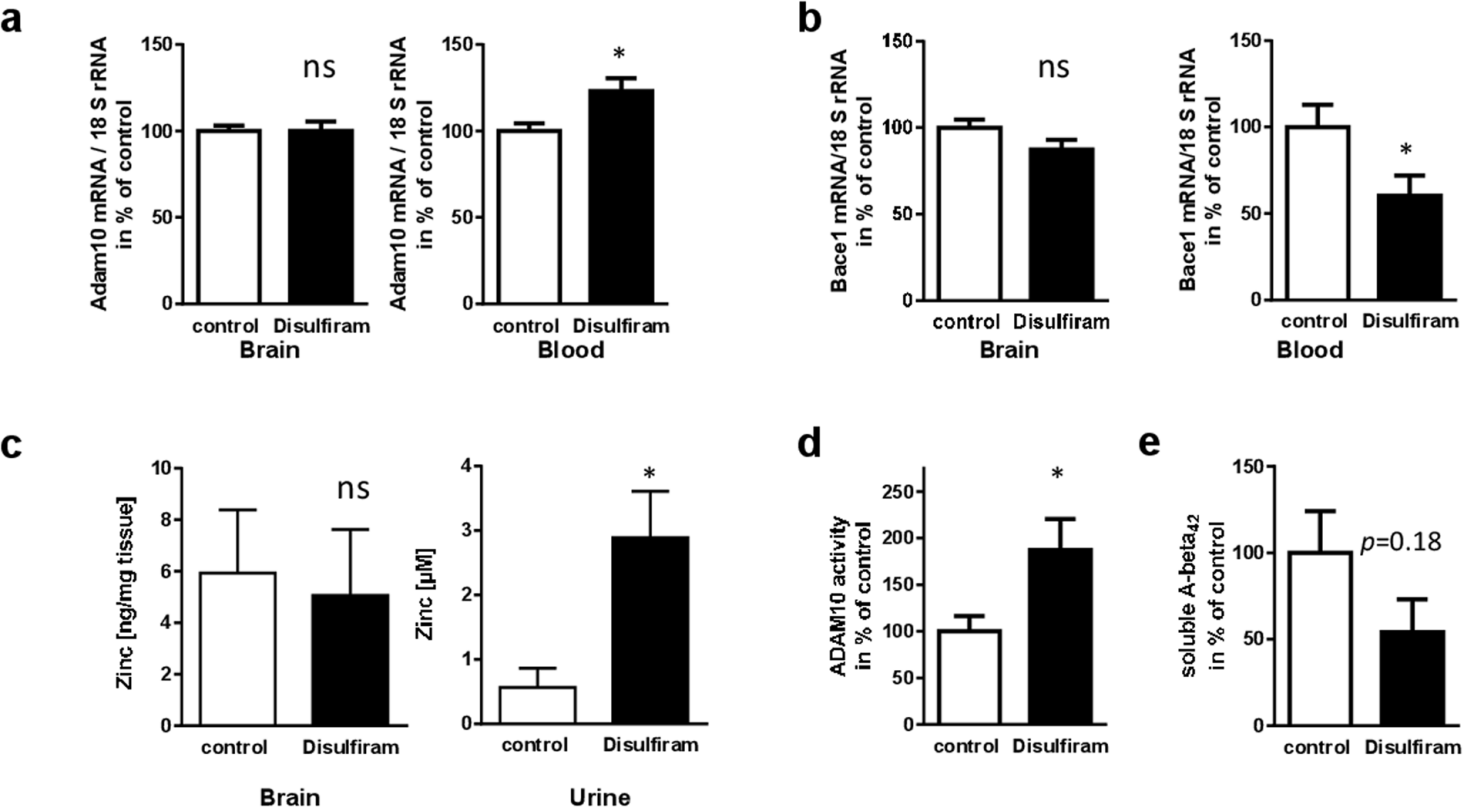
Impact of disulfiram on pathological hallmarks in AD model mice. Female 5xFAD mice were treated for 2 days with disulfiram or DMSO as control. On day three they underwent behavioral assessment and/or sacrifice and sample collection. Adam10 (**a**) or Bace-1 (**b**) mRNA was quantified from total RNA preparation of brain and blood cells (n = 10 animals per group for Adam10, n = 8 for Bace-1). RNA amount was normalized to 18 SrRNA levels and is presented as mean + SEM. (**c**) Quantitation of zinc concentration in brain homogenates or urine was performed using a fluorimetric assay (n ≥ 8 per group). Values are presented as mean + SEM. (**d**) Adam10 activity was measured by using a pro-fluorescent substrate. Slopes of the enzymatic reaction were assessed over 35 min and are presented as % of values (mean + SEM) obtained for control-treated animals (n = 7 per group). (**e**) Soluble A-beta_42_ peptides were quantified using a commercial ELISA assay (n = 4 animals per group). Values are presented as mean + SEM in % of values of control-treated animals. Statistical analysis: unpaired Student’s t-test (***p < 0.001; *p < 0.05; ns, p > 0.05).

To ascertain whether disulfiram also exhibits therapeutic potential *in vivo*, Alzheimer model mice (5xFAD) were treated for two days with the drug (52 mg/kg per day). This represents the human equivalent dosage (HED) calculated as described previously^24^ for a daily dosage of 250 mg in an adult weighing 60 kg (maintenance dose in alcohol dependent patients: 200 to 500 mg/day). Using the full HED, resulted in gastrointestinal difficulties in the mice (diarrhea, apathy, changes in gut tissue) and therefore it was decided to reduce the daily dosage to 26 mg/kg. This abolished undesired side effects and mice showed no signs of gastrointestinal impairment (see also Suppl. Figure 3). After two days of treatment with this comparably low dose, no increase of Adam10 mRNA in the brain was observed on the third day (Fig. 5a). Interestingly, Adam10 mRNA quantitation in blood cells revealed a disulfiram-dependent induction to 123% of control-treated animals. This was accompanied by decreased amounts of Bace-1 mRNA levels (60% of control; Fig. 5b) that were also only observed in blood but not in brain. This could indicate that the mechanism of regulation observed in cell culture experiments is different to that in the murine brain, that mRNA kinetics do not allow detection of elevated Adam10 and decreased Bace-1 mRNA 24 h after the last injection, or that disulfiram was administered in too low a concentration. In order to understand whether disulfiram is able to penetrate the brain in sufficient amounts and if it was systemically effective, zinc concentrations in brain tissue and in urine were measured. The chelator disulfiram should lead to an increase of its urinary excretion (in analogy to data on nickel^25^). Zinc levels remained relatively unaffected within the brain (Fig. 5c). However, urinary excretion of zinc was drastically elevated to about 300% of control animals. Therefore, disulfiram acts in a systemic manner: however, its concentration within the brain might have been too low or its effect might already have been reversed due to chemical conversion^26^ at the chosen time-point of investigation (24 h after the last injection). Because the half-life time of the Adam10 protein is rather long (72 h^27^) and increased amounts of the enzyme might still persist 24 h after the last drug administration, we nevertheless decided to analyze its catalytic activity in brain tissue: Adam10 activity was nearly doubled in brain material derived from disulfiram-treated mice as assessed by a pro-fluorescent substrate cleavage assay (Fig. 5d). Moreover, the amount of soluble A-beta_42_ was found to be decreased (Fig. 5e) although not reaching statistical significance in the small group of investigated animals.

**Figure 6:**
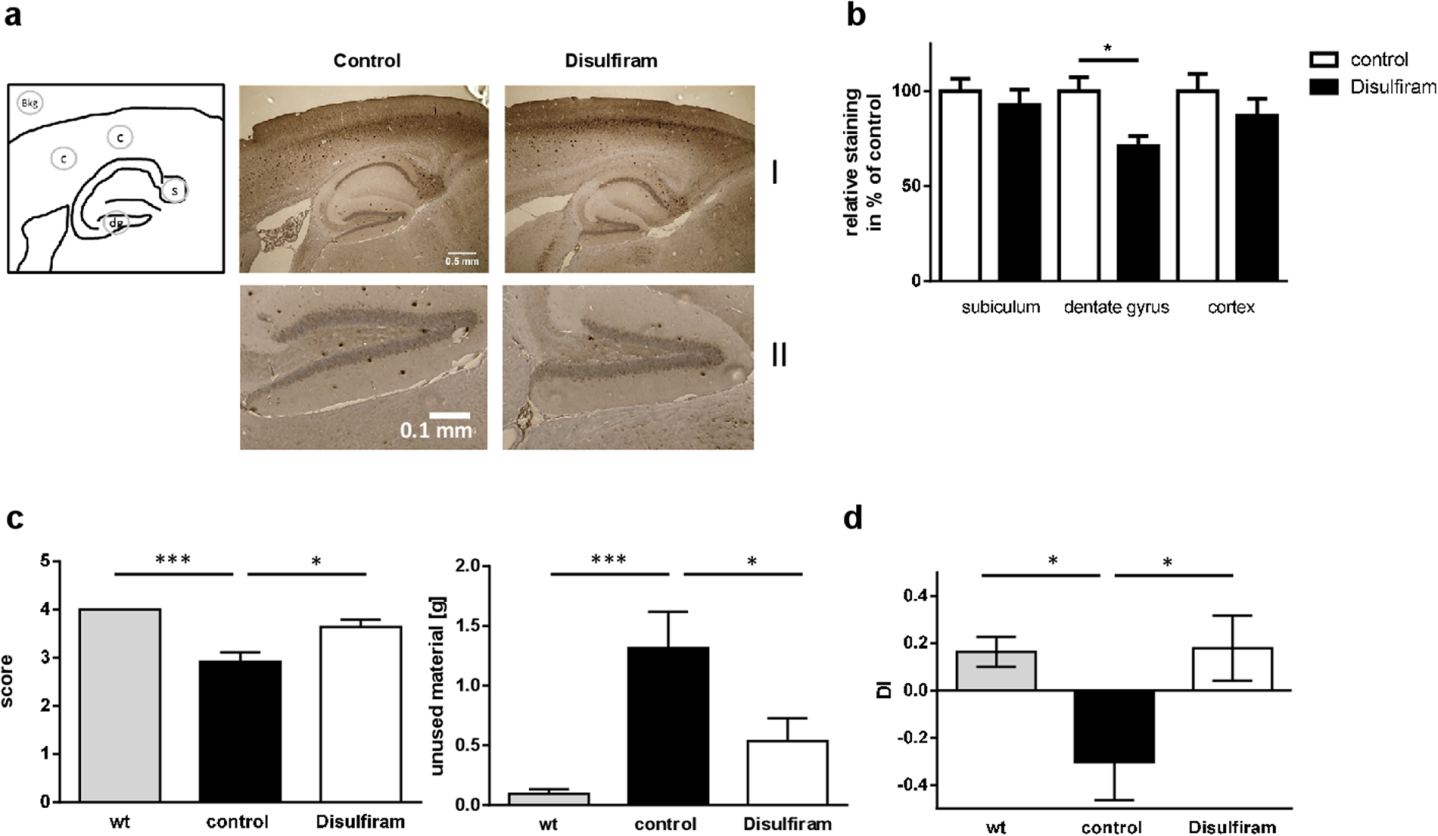
Influence of disulfiram on A-beta depositions and behavior in AD model mice. (**a**) Deposition of APP-cleavage products derived from transgenic human APP was assessed in brain slices of mice (description of treatment: see Fig. 4) by staining with antibody 6E10. Two cortical regions (c), subiculum (s) and dentate gyrus (dg) were used for densitometric quantitation from two slices per animal and corrected by a same-size background area as indicated in the scheme (I: whole area; II: magnification of dentate gyrus). Values are given as mean + SEM (**b**), n = 6 for control and 5 for disulfiram-treatment). (**c**) In the morning of day three, nest-building performance of the animals was determined by a scoring and also by weighing material that was not incorporated in the nest (n ≥ 7 per group). (**d**) Learning capability was tested in the mice by a novel object recognition paradigm performed in a Y-maze (n = 8 per group). Discrimination index is presented as mean ± SEM. Statistical analysis: unpaired Student’s t-test (***p < 0.001; *p < 0.05).

To assess potential pathomechanistic relevance of disulfiram-treatment in the Alzheimer mouse model brain slices of mice treated by two daily injections of disulfiram (26 mg/kg/day) were stained for A-beta depositions with antibody 6E10 (Fig. 6a I). Representative cortical regions and subiculum displayed only non-significant decrease in staining signals that represent oligomeric as well as plaque-deposited A-beta peptides. The dentate gyrus, on the contrary, showed a reduced staining intensity and plaque number in mice treated with disulfiram (Fig. 6a II and b; 71% of control).

We conducted two behavioral tests that rely on hippocampal integrity^28,29^ to investigate a potential beneficial effect of the short-term treatment with disulfiram on the AD model mice. In the nest building test, 5xFAD mice showed an impaired performance as compared to wild type mice as indicated by a lower score and higher amount of nesting material which was not incorporated in the nest (Fig. 6c; both mouse groups, wt and control, were injected with the solvent). AD model mice treated with disulfiram showed significantly increased nest building ability as measured by the score and amounts of integrated nesting material as compared to solvent-treated AD model mice. Additionally, the impairment of the 5xFAD mice observed in the novel object recognition test (Fig. 6d) in comparison to wild type mice was totally rescued upon treatment with disulfiram.

### Induction of ADAM10 in blood cells by clinical treatment with disulfiram

**Figure 7:**
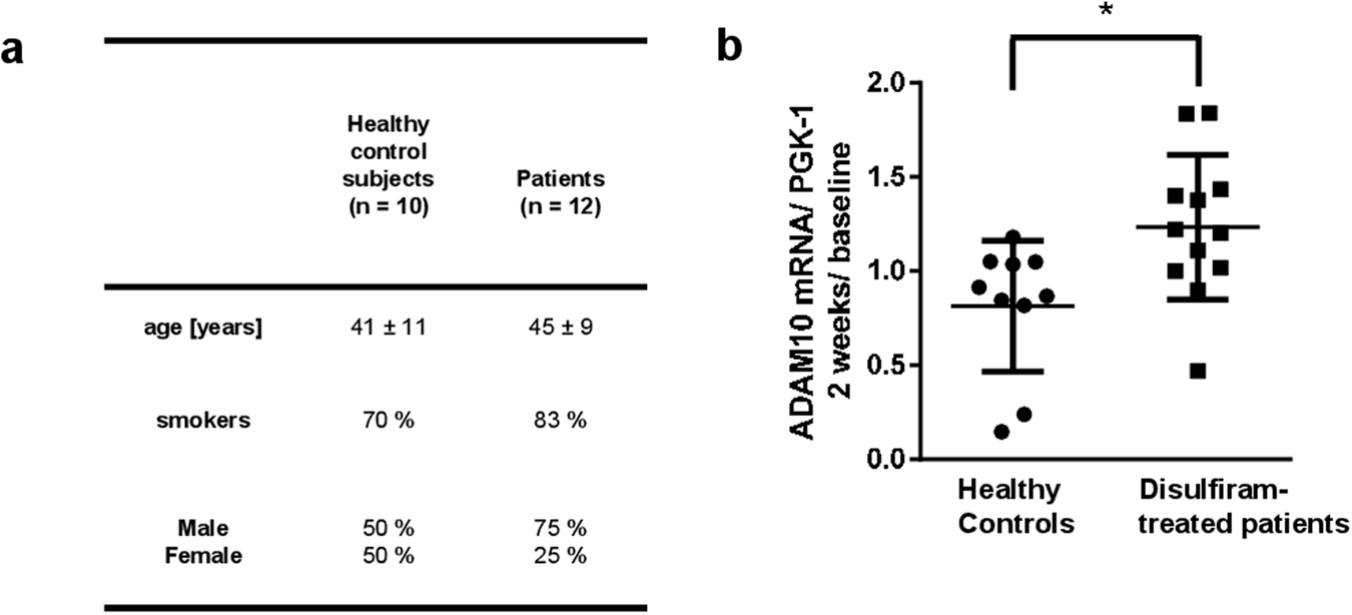
Longitudinal ADAM10 mRNA measurement in untreated healthy controls and in alcohol addicted patients treated with disulfiram. (**a**) Two groups of subjects were included in the investigation: healthy controls and alcohol dependent subjects. Both groups were matched for age and smokers rate. The gender rate differs significantly between the groups but represents normal ratios found in the healthy as well as the alcohol dependent part of the population (28). The alcohol dependent subjects were treated with disulfiram following the normal treatment schedule of the clinical centres (Department of Psychiatry and Psychotherapy, Mainz or CIMH, Mannheim). From both groups blood was sampled by venipuncture on day 1 and approx. 17 days later. One patient was excluded from the investigation due to lack of compliance and extremely extended time point for the second blood draw (3 months). (**b**) ADAM10 mRNA level was quantified from total RNA preparations in healthy controls and alcoholic dependent patients treated with disulfiram and is presented as the ratio between values obtained after ca. two weeks and at baseline (RNA amount was normalized to PGK-1 RNA levels). Mean and SEM are indicated. Statistical analysis: unpaired Student’s t-test (*p < 0.05).

Disulfiram is used in alcohol dependent patients as a relapse-preventing treatment. This allows an analysis in a natural clinical setting of the capacity of disulfiram to induce ADAM10 in human peripheral blood cells (for demographics see Fig. 7a). Blood was sampled at two time points: before the start of treatment (baseline) and after approximately two weeks (17 ± 3 days) of treatment. As stability of ADAM10 expression in blood cells over time has not been investigated so far to our knowledge, we also included a healthy control group without treatment and analyzed ADAM10 expression over a time frame of two weeks. While most healthy subjects displayed an unaltered ADAM10 expression, the vast majority of the disulfiram-treated patients showed an increase of levels of ADAM10 mRNA (Fig. 7b). Induction of ADAM10 in humans in peripheral tissue by disulfiram treatment is thus feasible but efficacy and safety has to be tested in AD patients in the future.

## Discussion

Development of novel drugs for the treatment of Alzheimer’s disease has to face a variety of challenges such as late-life disease diagnosis, lack of well-understood therapeutic targets and additionally penetrance of the blood brain barrier. Drug re-purposing may offer an important strategy to accelerate process (as shown in^30^). As an example, the cancer drug AM-80 has been demonstrated to contribute beneficial therapeutic value in AD model mice^31,32^. However, efficacy in human patients has always to be verified because most AD model mice rely on genetic manipulation and inadequately represent the typical patient suffering from sporadic AD. In the case of acitretin, a drug prescribed for psoriasis^33^, we showed that it is not only an ADAM10 inducer in cell culture and mice, but also has a positive effect in mild to moderately affected human patients^21,34^. Here, we took an unbiased approach and analyzed a library of FDA-approved drugs for their potential to enhance ADAM10 expression. We identified disulfiram as one of the most promising candidates, resulting in lowering of BACE-1 promoter activity but also an increase in ADAM10 promoter activity. This is important as drugs that only aim at reducing A-beta peptide levels failed to show therapeutic effects in clinical trials published to date. Increasing the amount of sAPP-alpha might be a more reliable therapeutic strategy as this protein fragment has been shown to exert neurotrophic and neuroprotective properties in many publications. For example, recombinant sAPP-alpha protected primary hippocampal neurons from cell death via activation of the Akt-survival pathway^35^.

To our surprise, treatment of the 5xFAD Alzheimer model mice with disulfiram did not elevate Adam10 mRNA levels in the brain, although the murine Adam10 promoter, similar to the human promoter, could be induced by the drug in cell culture experiments. However, the intensity of the increase in promoter activity was much lower for the murine gene than for the human and the response necessitated higher drug concentrations. Measurement of the zinc chelating property of disulfiram in mouse brain tissue demonstrated no change of the zinc concentration within the brain parenchyma as a result of the treatment, while the urinary excretion was significantly increased. It is thus possible that the effective concentration of disulfiram in brain is insufficient to increase Adam10 expression in spite of the observed increase in Adam10 levels in blood. One of the metabolites of disulfiram, N-acetyl-S-(N,N-diethylcarbamoyl) cysteine (DETC-NAC), could be detected in the nucleus accumbens and the medial prefrontal cortex of rats treated with 200 mg/kg disulfiram intraperitonally^36^. However, the concentration reached in these brain areas was much lower (1.1 and 2.5 nM) compared with plasma levels (80 nM) 6 h after application. The injected concentration used in the study presented here was much lower to avoid toxic effects and possibly insufficient to increase ADAM10 transcriptional activity in the brain. Additionally, measurement of mRNA levels was conducted 24 h after the last injection and disulfiram is – due to the instability of its disulfide bond – is highly reactive and therefore subjected to fast degradation^26^. Nevertheless, an increase of Adam10 activity as well as a tendency for reduced soluble A-beta_42_ peptides could be demonstrated in the brain of 5xFAD mice treated with disulfiram. An effect on the proteases Adam10 and Bace-1 might indeed have occurred but was only visible at the protein level due to the relative long stability of the protein in contrast to the stability of the respective mRNAs^27,37^. In peripheral blood cells, however, the ratio of Adam10 and Bace-1 mRNA was affected in favor of Adam10. Besides the assumed beneficial effect in brain, ADAM10 has been reported to produce a truncation product of Trx1, thioredoxin 80 (Trx80), from monocytes^38^. This blood-derived proteolysis product has been detected in human brain and seems to be protective against aggregation of A-beta peptides. This fact, together with the *in vitro* observed aggregation-inhibiting property of disulfiram and its influence on Adam10 activity might be sufficient to explain the observed reduction in 6E10-positive staining within the dentate gyrus of the mice and the resulting increase in behavioral performance in two tasks that at least partly depend on hippocampal function. Nevertheless, we cannot exclude that additional effects on the immune system, on mitochondrial function or on other relevant molecules contribute to the beneficial outcome. For example, our own study revealed that disulfiram has the capability to lower GSK3β activity *in vitro* which might result in altered Tau posttranslational modification also *in vivo*.

Along with the clinical use of disulfiram in the treatment of alcohol dependence, side effects of cognitive performance such as drowsiness, impaired attention and memory impairment have been described^39,40^. In addition, cases of psychosis have been associated with high dosage in alcohol dependent patients^41,42^ and worsening of symptoms in schizophrenic patients^43^. An early investigation on intellectual function of patients treated with disulfiram reported no impairment^44^, while Prigatano and colleagues demonstrated a reduced improvement in neuropsychological functioning in disulfiram-treated patients versus a milieu treatment program group three weeks after acute alcohol detoxification^45^. In sum, disulfiram might act as a deleterious substance on cognitive functioning in the context of alcohol abuse and patients with increased vulnerability (personal or familial antecedents of psychosis). However, in healthy prisoner volunteers, high dosage of the drug (0.5 g/day to 1.5 g/day) also resulted in impaired immediate memory and e.g. color naming^43^. Using a more typical dosage of 0.5 g/day, Peeke and colleagues reported in non-alcoholic volunteers over two weeks on a psycholocigal test battery very few significant differences between baseline and disulfiram-treated sessions. Interestingly, these were in the opposite direction to that expected and demonstrated improvement under disulfiram-medication (DSS task, confusion assessment^46^). Predicting a beneficial outcome for Alzheimer patients under therapy with disulfiram thus remains difficult, even in the light of the encouraging data from the mouse model. In regard to the clinical study presented here, it also has to be considered that stability of ADAM10 over the two weeks of investigation in untreated condition has only been demonstrated in healthy controls. In the alcohol-dependent patients treated with disulfiram, the pre-post-comparison revealed an increase of ADAM10 expression. However, it cannot be excluded that the condition of non-alcohol intake enforced by disulfiram alone might also affect ADAM10 levels. Another aspect to be considered are the effects on the cholinergic system that have been reported for disulfiram, for example effects on the autonomous nervous system in rodents. A decrease in the histochemical reactivity for acetylcholine-esterase was observed in the nerve plexuses of the gut wall in rats treated with 220–580 mg/kg for 1–3 weeks^47^. In the treatment presented here which included a paradigm with lower dosage, no significant changes in acetylcholine-esterase levels in gut or brain tissue were observed (shown in Suppl. Figure 3).

Nevertheless, a 15 days treatment of rats with disulfiram, even at low doses (50 mg/kg/day) showed a selective elevation of acetylcholine in the hippocampus of the animals^48^. The assumption that acetylcholine deficit is one of the major drivers of cognitive decline in Alzheimer’s disease underpins the clinic treatments used today for Alzheimer patients, namely acetylcholine esterase inhibitors such as Donepezil (e.g. reviewed by^49,50^). In our study we did not test the release or synthesis of acetylcholine, but such an effect could clearly contribute to the observed amelioration of cognitive deficits. Such an add-on effect might also be contributed by effects on structural properties of the amyloid precursor protein or its cleavage products. It has been shown that sAPP-alpha production increased upon disulfiram application by inhibiting dimerization of the APP ectodomain in 7W-CHO non-neuronal cells^51^. In addition, Nagai and Ito reported that inhibition of hydrogen peroxide production via disulfiram attenuated the increase in Abeta1-42 in the lens capsule-epithelium of a rat cataract model^52^.

In conclusion, disulfiram may influence AD pathology in multiple ways, resulting in an increase of functional behavior at least in mice at low doses. Low compliance by demented patients can be partially circumvented as disulfiram can be administered by subcutaneous implantation^53^. Development of novel encapsulated or nanoparticle-assisted forms of the drug^54,55^ might additionally help in the establishment of a potential therapy. However, safety in AD patients and impact on cognition have to be investigated in the future.

## Materials and Methods

### Material

The FDA approved drug library (Enzo) was kept at −80 °C. Aliquots were prepared on clear 96 well plates using the Liquidator (Qiagen). Disulfiram for further analyses was purchased from Sigma.

### Cell culture

The human cell line SH-SY5Y was maintained at humidified air (95%), 5% CO_2_, 37 °C, and cultured in DMEM/F12 (Life Technologies, Darmstadt, Germany) supplemented with 10% FCS and 1% Glutamine (GE Healthcare, Piscataway, NJ, USA).

### Toxicity assay

Cells were seeded at a density of 45 000 cells per well of a white glass-bottom 96 well plate (Greiner Bio-OneGmbH, Frickenhausen, Germany) in OptiMEM (Life Technologies, Darmstadt, Germany) and incubated with the substances of the FDA-approved library. Cell viability was assessed using the Cell Titer Glo Assay (Promega). Concentrations that were found toxic (decrease in viability of >20% in comparison to solvent control) or pro-proliferative (increase in viability >20%) were reduced until appropriate.

For measuring a potential protective effect of disulfiram on A-beta treatment of cells, SH-SY5Y cells were seeded at a density of 38 000 cells per well of a 96 well plate (Greiner Bio-OneGmbH, Frickenhausen, Germany) in culture medium supplemented with solvent (DMSO), human A-beta_42_ peptides (2.5 µM, Anaspec) or peptides combined with 2.2 µM disulfiram for 48 h. Subsequently, MTT reagent was added (5 µl of 5 mg/ml stock solution) and viability assessed as described before^56^.

### Reporter gene assay

The dual luciferase reporter assay was performed as described previously^57^. In brief, a reporter vector was used for simultaneously detecting human BACE-1 promoter activity and human ADAM10 promoter activity with the dual luciferase reporter kit (Promega) after incubating the cells for 48 h. Substances were diluted following results from the toxicity assay and the assay was at least performed three times independently for each substance. Both promoter sequences as well as the single reporter vector for ADAM10 promoter are described in a former publication^57^. The reporter vector for murine ADAM10 promoter activity has also been reported previously^21^.

### Western blotting

Cells were seeded on 12-well plates at a density of 500 000 per well and incubated with the substances as indicated for 48 h. Cell supernatant from a 5 h secretion period was collected and soluble proteins precipitated via TCA^21^. Cells were harvested in PBS, protein content determined, and half of the supernatant or 20 µg proteins subjected to SDS polyacrylamide gel electrophoresis. Proteins were blotted onto nitrocellulose and blocked with 0.2% I-Block (Thermo Fisher Scientific) solution including 0.05% Tween20. Primary antibodies were as follows: ADAM10 rabbit polyclonal antibody (Merck, Darmstadt, Germany), ADAM17 rabbit polyclonal antibody (Chemicon, Merck), BACE-1 rabbit monoclonal antibody (D10E5, Cell Signaling Technology, Danvers, MA, USA), APP N-terminal mouse monoclonal antibody (for detection of sAPP-alpha, 6E10, Covance, Madison, WI, USA), anti-sAPP-beta (Covance, Madison, WI, USA) or APP C-terminal antibody^58^. As a loading control, GAPDH was detected (14C10, Cell Signaling, Danvers, MA, USA). Blots were incubated with respective secondary antibody coupled with horseradish peroxidase (Thermo Scientific, Karlsruhe, Germany) and signals obtained by applying SuperSignal West Femto chemiluminescent substrate (Thermo Scientific, Karlsruhe, Germany) were captured using a CCD-camera imaging system (Raytest, Straubenhardt, Germany).

### Amyloid-beta aggregation assay

The amyloid aggregation assay was conducted in 50 mM Tris/150 mM NaCl (pH7.2) supplemented with 125 µM Thioflavin T (Sigma) and 0.1 mg/ml human AggreSure beta-Amyloid (1–42) (Anaspec) in black 384 well plates. Fluorescence was measured in a Fluostar Omega (BMG Labtech, Cary,NC, USA) (Ex/Em = 440/484 nm) to analyze A-beta aggregation.

### GSK3-beta activity assay

The luminescent GSK3-beta assay was performed as recommended by the vendor (Promega).

### Animals

Male 5xFAD mice (Jackson Lab) were crossbred with C57BL/6 J female mice for maintenance. Non-transgenic female offspring was used as control. All animals were group-housed in cages in a 12 h light/dark cycle with food and water available ad libitum unless otherwise stated (three to five animals/cage). All procedures were performed in accordance with the European Communities Council Directive regarding care and use of animals for experimental procedures and were approved by local authorities (Landesuntersuchungsamt Rheinland-Pfalz; approval number G14-1-087).

### Disulfiram treatment

Mice aged 3 months were assigned randomly into the control group (solvent, DMSO) or the disulfiram group (26 mg/kg per day). Disulfiram (stock solution of 100 mg/ml, kept in aliquots at −20 °C) was diluted freshly in DMSO to 0.8 mg/150 µl injection solution. Animals were injected i.p. at a fixed time in the morning of two consecutive days.

### Behavioral procedures

For the assessment of nest building capability, animals were single-caged and habituated to the nesting material used for scoring (Sizzle Pet Nesting Material, Claus GmbH, Germany; 10 g per cage) for 24 h in the presence of their former nest building material (paper towel). Animals then received fresh nesting material for three days. Subsequently, animals were deprived for 24 h to enhance nest building motivation and also received their first injection. On day six, mice were injected the second time and cages were supplemented with fresh nesting material to allow nest building for overnight. The following morning, quality of the nest was scored as described by Deacon^59^ (e.g. 0: no nest built; 5: perfectly closed dome built). Additionally, the nesting material that has not been introduced into the nest was weighed.

For the novel object recognition (NOR) test, mice were injected on day one and two. On day two they were additionally habituated for 6 min to the Y-maze which was used for the NOR test. On the third day, mice were represented in a first session for 6 min to two identical objects in two arms of the Y-maze. Two hours later, one of the objects was replaced by a novel object and mice allowed to explore the maze for another 6 min. A computerized video system registered moving-path and presence in the defined areas (non-displaced (NDO) and displaced object (DO)) automatically (hardware consisted of an IBM-type AT computer combined with a video digitizer and a CCD video camera). The software used for data acquisition and analysis was EthoVision XT release 8.5 (Noldus Information Technology, Utrecht, Netherlands). Analysis of the data was performed using the discrimination index (time (DO-NDO)/ time (DO + NDO)).

### Clinical study

Human healthy controls and patients were recruited from the Clinic of Psychiatry and Psychotherapy (Mainz) or from the CIMH (Mannheim). Exclusion criteria for the patients were a diagnosed Alzheimer dementia and a treatment with disulfiram less than four weeks before baseline assessment. Patients were included that were treated with disulfiram for alcohol relapse prevention. Exact treatment schedule was chosen by the medicating physician. In general, a dosage of 1500, 1000 and 500 mg is used for the first three days, and then a dose of 500 mg three times a week is prescribed. Patients that were compliant for 14 days were included in the study. All patients gave informed consent for study participation; the study has been approved by local authorities (Landesärztekammer Rheinland-Pfalz), and is registered with clinical trials.gov (NCT03212599). All experiments were performed in accordance with relevant guidelines and regulations.

### Tissue dissection and sample collection

Mice were anaesthetized with isofluran and sacrificed by decapitation. Truncal blood was mixed with RNA later (Qiagen, Hilden) and urine was collected. One brain hemisphere was drop fixed in 4% formaldehyde for IHC and the other hemisphere immediately cut in cubes and submerged in RNA later for preservation or snap frozen for zinc measurements and assessment of Acetylcholine esterase levels by the method of Ellman (see Suppl. Figure 2). In addition, sections of the gut were taken to assess Acetylcholine esterase levels. All samples were stored at −80 °C before usage despite IHC samples.

For human blood, samples were collected in a 2.5 ml EDTA monovette (Sarstedt, Nümbrecht, Germany), inverted three times, stored upright and processed within maximal 20 minutes after puncture. 0.5 ml blood was pipetted to 1.3 ml RNAlater and mixed by inverting. Samples were stored at −80 °C before further use.

### Zinc quantitation

Snap frozen hemispheres were grinded manually under nitrogen cooling and 1 mg material suspended in water used for assessment of zinc amount with the fluorogenic zinc quantification kit (Abcam). Values were corrected for background fluorescence calculated as ng/mg tissue wet weight. Urine was used undiluted and obtained values corrected for autofluorescence.

### A-beta ELISAs

SH-SY5Y cells were seeded on 12 well plates at a density of 500 000 cells per well and medium was exchanged 5 h after passage with medium containing 2.2 µM disulfiram or solvent (DMSO). 24 h later, cell supernatant was exchanged by 1 ml fresh drug-containing medium per well and secreted proteins were collected for 16 h. 200 µl of cell supernatant were subjected to human A-beta 1-x ELISA as recommended by the vendor (IBL).

Brain tissue grinded under nitrogen cooling was suspended in extraction buffer (100 mg/800 µl; 10 mM Tris HCl pH7.4; 100 mM NaCl, 1% TritonX100, 0.1% SDS, 1 mM EDTA, Protease-Inhibitor without EDTA (RocheMini complete)) and homogenized with a tissue mill (TissueLyzer, Qiagen, Hilden, Germany) and stainless steel beads (5 mm, Qiagen). The suspension was incubated on ice with vortexing every 10 min for 30 min, centrifuged (13 000 g, 4 °C, 10 min) and the supernatant diluted 1:200 in standard diluent buffer of the ELISA kit (Human Abeta_42_, Invitrogen, Camarillo, CA, USA). The assay was performed as recommended by the vendor.

### ADAM10 activity assay

SH-SY5Y cells were seeded on 24 well plates at a density of 260 000 cells per well and medium was exchanged 5 h after passage with medium containing 2.2 µM disulfiram or solvent (DMSO). After 48 h of incubation, cells were scraped and collected by centrifugation (13 000 g, 4 °C, 3 min). The cell pellet was resuspended in 20 µl assay buffer (AnaSpec) and incubated for 15 min on ice. 8 µl of cell suspension were supplemented with 2 µl DMSO or GM6001 solution in black 384 well plates.

Tissue was prepared as described for A-beta ELISA. The pellet from centrifugation was washed in 200 µl PBS with protease inhibitors. 50 µl of tissue suspension was pelleted (3 min, 3,000 g, 4 °C) and washed two-times in assay buffer provided by the vendor (100 µl, AnaSpec, Seraing, Belgium) with 10 min incubation on ice. 5 µl of tissue suspension was supplemented by 3 µl assay buffer and 2 µl of DMSO or GM6001 solution in black 384 well plates.

For both, cell suspension or tissue lysate, 10 µl of pre-warmed substrate solution were added and kinetics measured by a FluoStarOmega (BMG Labtech; 60 min measurement with measuring every 2 min after orbital shaking, 37 °C). Slopes of fluorescent signal-increase over time as measured by Em485 nm/Exc520 nm for non-inhibited reaction (DMSO) were corrected by values obtained from inhibited reaction (GM6001) to make sure that only substrate digestion by metalloproteinases is taken into account.

### Plaque analysis

IHC sections were prepared and stained with anti-APP antibody 6E10 (Covance) as described previously^56^. Two sections per mouse were used for densitometric analysis in a total magnification of 40 × : five areas were determined to be measured as shown in Fig. 6a. All areas were corrected for the value of the background area. For cortical tissue, two distinct areas were analyzed and integrated into a mean value. Experimenters were blinded for the treatment of the mice during analysis.

### Quantitative real-time PCR

Total RNA was extracted according to the manufacturer’s instructions (RNA II, Qiagen, Hilden). For human and murine blood, RNA was extracted with the RiboPure Blood Kit (Ambion) as recommended by the manufacturer. 20ng (mouse blood) or 100 ng RNA were used for quantitative Real-time PCR with the QuantiTectSYBRGreen Kit (Qiagen, Hilden) performed in a StepOnePlusCycler (Life Technologies, Darmstadt, Germany). Primers were as follows: Adam10 (QT00106351), Bace-1 (QT00493948), murine 18SrRNA (QT02448075), ADAM10 (QT00032641), PGK-1 (QT00013776). Levels of mRNA were normalized to the mRNA levels of the house keeping genes (18SrRNA/PGK-1) using a standard curve.

### Quantification and imaging

Quantitative analysis of Western blot chemiluminescent signals or chromogenic signals from IHC sections was carried out with AIDA image analyzer 4.26 software (Raytest, Straubenhardt, Germany). Microscopic pictures of the IHCs were acquired by an EVOS XL microscope (Life Technologies, Darmstadt, Germany). Scale bars were inserted by using ImageJ software^60^.

### Statistical analysis

For comparison of two groups, data were analyzed by Student’s t-test, followed by Bonferroni’s correction. One-way analysis of variance (ANOVA) was performed for three or more groups, followed by the LSD post hoc test. P-values < 0.05 were considered statistically significant and results were represented as mean ± SD (cell culture/*in vitro* experiments) or ± SEM (animal experiments/clinical study). Data analyses were performed using GraphPad Prism 6 (Graph Pad Software, La Jolla, CA, USA).

### Data availability statement

The datasets generated during and/or analyzed during the current study are available from the corresponding author on reasonable request.

## Electronic supplementary material

Supplementary material
