# Supplementary material for: Identification of disulfiram as a secretase-modulating compound with beneficial effects on Alzheimer’s disease hallmarks
Source: Sci Rep. 2018 Jan 22;8:1329. doi: 10.1038/s41598-018-19577-7 (PMC5778060; doi:10.1038/s41598-018-19577-7)
Supplement: Supplementary file 1 — Supplementary material [file 41598_2018_19577_MOESM1_ESM.pdf]

Identification of disulfiram as a secretase-modulating compound with beneficial effects on Alzheimer's disease hallmarks

Dr. rer. nat. Sven Reinhardt, Dipl.-Biol. Nicolai Stoye, Dr. med. Mathias Luderer, Prof. Dr. Falk Kiefer, Prof. Dr. Ulrich Schmitt, Prof. Dr. Klaus Lieb, Dr. rer. nat. Kristina Endres

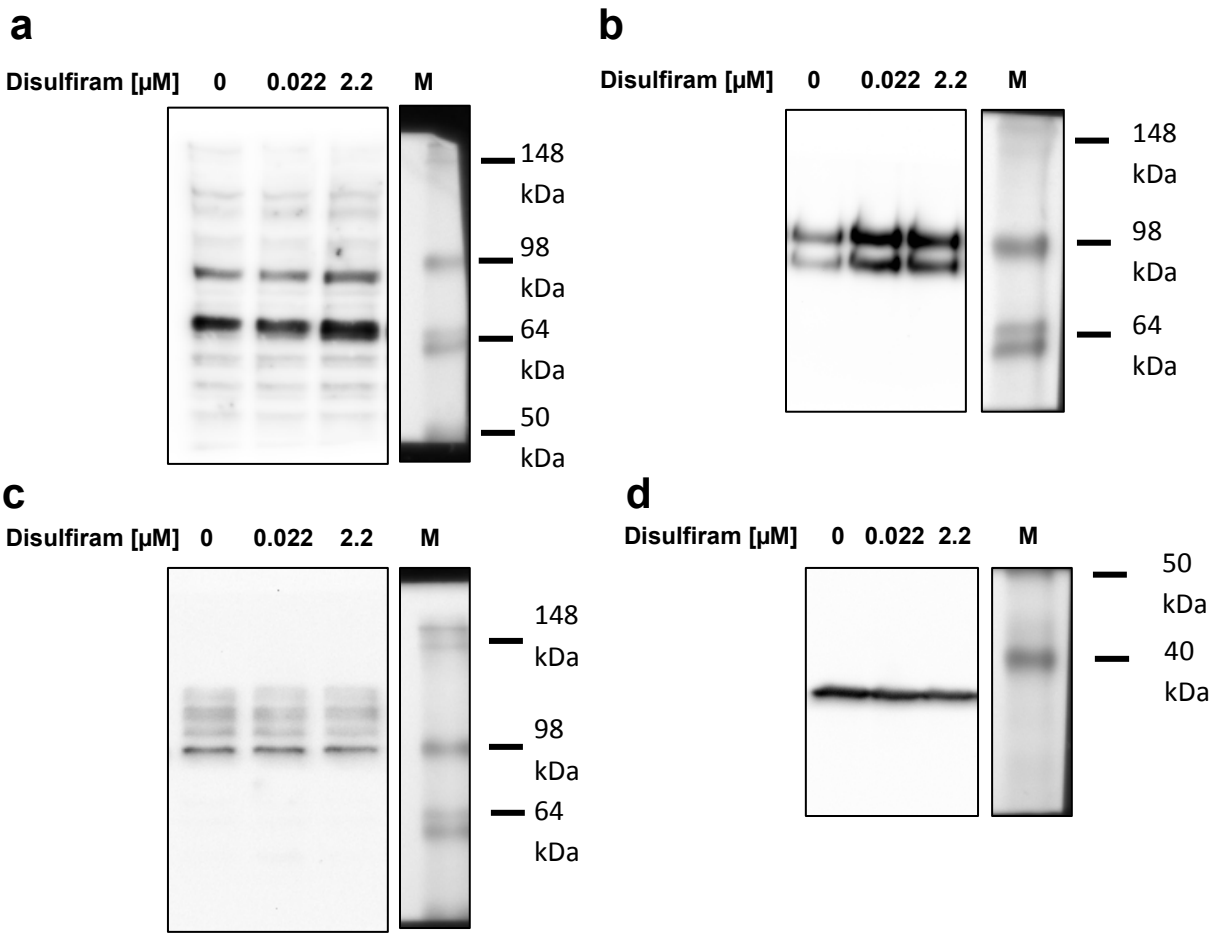

Suppl. Fig. 1 Examples of full-length blots from Fig. 2 C

Western Blot pictures in Fig. 2 C have been cropped for being able to combine several blots in one picture. Here, examples of full-length blots for each protein-detection are given where possible. Blots with cell lysates were cut at a height between 50 and 40 kDa to use the upper blot part for detection of ADAM10 or full-length APP and the lower blot part for detection of GAPDH. a) ADAM10; b) sAPP-alpha; c) APP; d) GAPDH. The lane with the marker (M) has been added to indicate molecular weights.

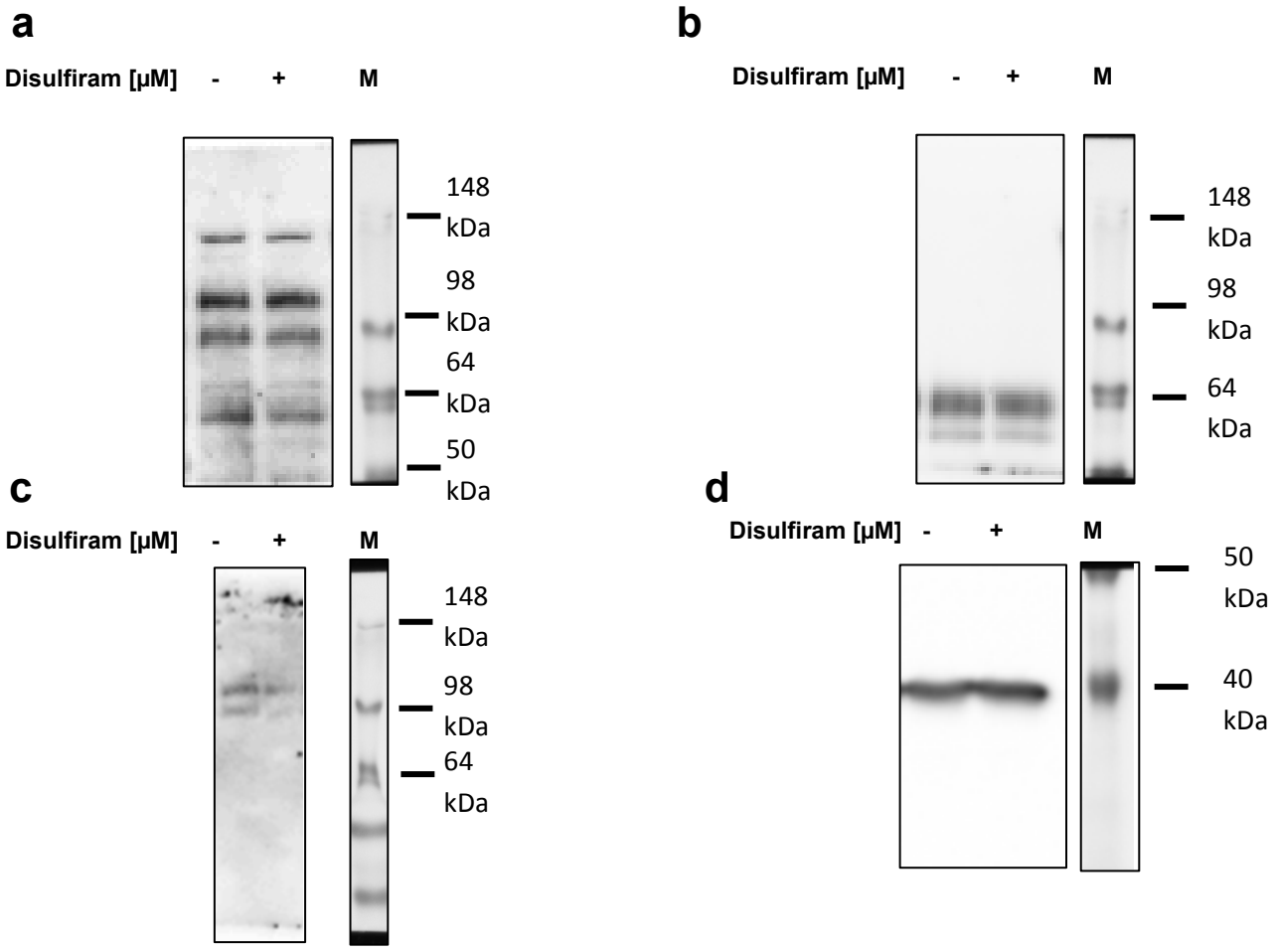

Suppl. Fig. 2 Examples of full-length blots from Fig. 3
Western Blot pictures in Fig. 3 have been cropped for presentation. Exemplary pictures of full-length blots for each protein-detection are given where possible. Blots with cell lysates (BACE1, TACE) were cut at a height between 50 and 40 kDa to use the upper blot part for detection of the proteinases and the lower blot part for detection of GAPDH. a) TACE; b) BACE1; c) sAPP-beta; d) GAPDH. The lane with the marker (M) has been added to indicate molecular weights.

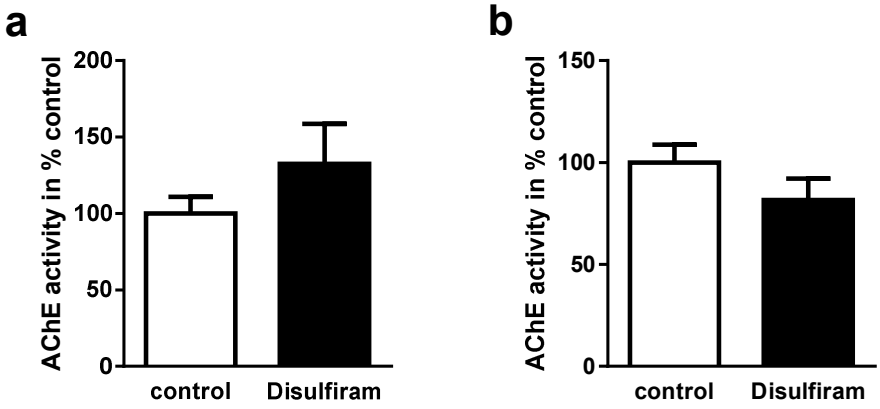

Suppl. Fig. 3 Acetylcholine esterase activity in brain and ileum of disulfiram-treated AD model mice
Tissue from mice treated for two days with disulfiram or DMSO as control were sacrificed and brain and ileum tissue collected (ileum tissue was mechanically purified and washed with PBS). Homogenates of tissue samples were prepared in potassium phosphate buffer and diluted aliquots used for measuring AChE activity with Ellman‘s reaction (a: brain; b: ileum; n≥9 per group). Values are given as mean + SEM, statistical analysis was conducted by unpaired Student‘s t-test (Welsh correction for analysis of data from brain; ns, $p>0.05$).

Suppl. Table 1 FDA-approved drugs tested for ADAM10 and BACE-1 promoter regulatory effect

Substances were initially tested for potential toxic/ proliferative effects in SH-SY5Y cells and subsequently diluted to obtain the final concentration in the dual promoter assay. 13 drugs were excluded because of remaining toxic effects and are therefore not included in the list.

Nr.	Drug	final conc. [µM]	Nr.	Drug	final conc. [µM]	Nr.	Drug	final conc. [µM]
1	Moroxydine HCl	3,15	101	Sulpiride s(-)	1,91	201	Denbufylline	2,04
2	Clindamycin HCl	1,54	102	Diphenhydramine HCl	2,56	202	Miltefosine	1,60
3	Felbamate	2,74	103	Promethazine HCl	2,30	203	Latanoprost	1,51
4	Cyclosporin a	0,54	104	Ranitidine HCl	2,08	204	Ouabain	0,02
5	Donepezil HCl	1,57	105	Naltrindole HCl	1,58	205	Clopamide	1,89
6	Lincomycin	1,61	106	Idazoxan	3,20	206	Molsidomine	2,70
7	Lomofungin	2,08	107	Spiperone	1,65	207	Pravadoline	1,73
8	Mycophenolic acid	0,08	108	Epinephrine-(+)-tartrate l(-)	3,86	208	Tranilast	2,00
9	Novobiocin Na	1,07	109	Xylazine HCl	2,97	209	Alfuzosin	1,68
10	Rapamycin	0,71	110	Norepinephrine-(+)-tartrate (-)	2,05	210	Bromocriptine mesylate	1,00
11	Spectinomycin	1,97	111	Quetiapine fumarate	0,74	211	Clozapine	2,00
12	Bepridil	1,58	112	Alprenolol HCl	2,62	212	Clothiapine	1,90
13	Amiodarone	1,01	113	Imipramine HCl	2,33	213	Acitretin	2,00
14	Nicardipine	1,27	114	Amoxapine	2,08	214	Remoxipride	1,76
15	Pimozide	1,42	115	Mesulergine HCl	1,80	215	Calcifediol	1,63
16	Loperamide	1,27	116	Amfebutamone	2,73	216	Calcitriol	1,57
17	Fluspirilene	1,37	117	Metoclopramide HCl	2,18	217	Ketoconazole	1,23
18	Tolbutamide	2,42	118	Naltriben methanesulfonate hydrate	1,28	218	Oxatomide	1,53
19	Pinacidil	2,66	119	Vitamin a (acetate)	1,99	219	Disodium cromoglycate	1,28
20	Glipizide	1,47	120	Levallorphan tartrate	2,31	220	Capsaicin	2,14
21	PhentolamineHCl	2,06	121	Nalbuphine HCl	1,83	221	Dexamethasone	1,67
22	Quinine	1,81	122	Carbamylcholine Cl	4,44	222	Dipyridamole	1,30
23	Flufenamic acid	2,32	123	Carbamyl-beta-methylcholine Cl	4,05	223	Ethacrynic acid	2,16
24	Propafenone	1,91	124	Butyrylcholine Cl	3,75	224	Indomethacin	1,83
25	Phenytoin	2,59	125	Famotidine	1,94	225	Naproxen	2,84
26	Procainamide	2,40	126	Terfenadine	1,39	226	Clofibrate	2,69
27	Lidocaine	2,41	127	Isoniazid	4,77	227	Ibuprofen	3,17
28	Flecainide	1,38	128	Ticlopidine HCl	2,48	228	Benserazide HCl	2,54
29	Rosiglitazone maleate	1,38	129	Amorolfine	2,06	229	Debrisoquin sulfate	3,73
30	Amantadine HCl	3,48	130	Clemastine fumarate	1,90	230	Actarit	3,38
31	Oxymetazoline HCl	2,51	131	Vardenafil	1,34	231	Bumetanide	1,79
32	Ifenprodil	2,01	132	Linezolid	1,94	232	Neomycin sulfate	1,06
33	Naftopidil 2HCl	1,67	133	Docetaxil	0,01	233	Auranofin	0,02
34	Prazosin HCl	1,56	134	Olopatadine	1,94	234	Captopril	3,01
35	Clonidine HCl	2,45	135	Manidipine	1,07	235	Docebenone	2,00
36	Guanabenz acetate	2,83	136	Tolcapone	2,39	236	Tranylcypromine	4,91
37	Rilmenidine hemifumarate	3,63	137	Gestrinone	2,12	237	Piroxicam	1,97
38	Buphenine	1,95	138	Olmesartan	1,17	238	Moxifloxacin HCl	1,49
39	Yohimbine HCl	1,67	139	Nisoldipine	1,68	239	Troleandomycin	0,01
40	Dihydroergotamine mesylate	1,12	140	Lacidipine	1,43	240	Carbidopa	2,89
41	Nicergoline	1,35	141	Olanzapine	2,09	241	Nimesulide	2,12
42	Emtricitabine	2,64	142	Lovastatin	1,62	242	Ketoprofen	2,57
43	Betaxolol HCl	2,13	143	Lamotrigine	2,55	243	Meloxicam	1,86
44	Practolol	2,45	144	Azathioprine	2,36	244	Terbinafine HCl	2,24
45	Timolol maleate (s)	2,07	145	Sildenafil	1,38	245	Sodium phenylbutyrate	3,51
46	Xamoterol hemifumarate	1,93	146	Atovaquone	1,78	246	Ergothioneine	2,85
47	Clenbuterol	2,36	147	Sertaconazole	1,49	247	Ambroxol	1,73
48	Procaterol HCl	2,25	148	Butenafine	2,06	248	Idebenone	1,93
49	Salbutamol sulfate	2,73	149	Dorzolamide	2,01	249	Mevastatin	1,67
50	Pindolol	2,63	150	Escitalopram	2,01	250	Simvastatin	1,56
51	Cimaterol HCl	2,98	151	Eprosartan	1,54	251	Suramin sodium	0,51
52	Dobutamine HCl	2,17	152	Sodium phenylacetate	4,13	252	Goserelin acetate	0,51
53	Pronethalol HCl	0,11	153	Ozagrel	2,86	253	Guaiacol	5,26
54	Sotalol HCl	2,40	154	Entacapone	2,14	254	Retinoic acid	2,18
55	Maprotiline HCl	2,36	155	Bopindolol malonate	1,72	255	Troglitazone	1,48
56	Nisoxetine HCl	2,41	156	Guanfacine HCl	2,31	256	Bezafibrate	1,81
57	Canthaxanthin	1,16	157	Tizanidine HCl	2,58	257	Raloxifene HCl	0,06
58	Pilocarpine HCl	3,14	158	Meglumine	3,35	258	Etoposide	0,04
59	Ipratropium Br	1,97	159	Propranolol HCl s(-)	2,21	259	Mitomycin c	0,08
60	Pirenzepine 2HCl	1,86	160	Carvedilol	1,61	260	Puromycin 2HCl	1,20
61	Telenzepine 2HCl	1,76	161	Cerivastatin	0,06	261	Delavirdine mesylate	0,06
62	Tropicamide	2,30	162	Aclarubicin	0,02	262	10-hydroxycamptothecin	0,04
63	Pancuronium Br	1,14	163	Flumazenil	2,15	263	Doxorubicin HCl	0,05
64	Ivermectin	0,75	164	Fenretinide	1,67	264	Cetirizine 2HCl	1,42
65	Physostigmine sulfate	2,37	165	Gefitinib	1,46	265	Lapatinib	0,04
66	Nialamide	2,19	166	Ibudilast	2,84	266	Methyl salicylate	0,17
67	Haloperidol HCl	1,74	167	Imatinib	1,32	267	Pioglitazone	1,83
68	Piribedil HCl	2,19	168	Montelukast	1,11	268	Pranlukast	1,36
69	Mepyramine maleate	2,29	169	Deprenyl	2,92	269	Rivastigmine	2,61
70	Trans-triprolidine HCl	0,05	170	Vatalanib	1,88	270	Rofecoxib	2,08
71	Cimetidine	2,59	171	Fumagillin	1,43	271	Ergotamine D-tartrate	1,12
72	Tiotidine	2,09	172	Exemestane	2,21	272	Sulindac	1,83
73	Zonisamide	3,08	173	Dinoprostone	1,85	273	Valproic acid	0,18
74	Zoledronic acid	2,40	174	Metformin	5,06	274	Calcipotriene	1,58
75	Naltrexone HCl	1,91	175	Anagrelide	2,55	275	Zafirlukast	0,01
76	zolmitriptan	2,15	176	Dofetilide	1,48	276	Zileuton	0,11
77	Memantine HCl	3,65	177	Erlotinib	1,66	277	Pentamidine	1,92
78	Aniracetam	2,98	178	Lobeline	1,94	278	Bambuterol	1,78
79	Riluzole HCl	2,79	179	Melatonin	2,81	279	Fentiazac	1,98
80	Propofol	3,67	180	Dinoprost	1,84	280	Mephenytoin	2,99
81	Mianserin HCl	2,47	181	HA1077	1,79	281	Diazoxide	2,83
82	Aminophylline	3,63	182	Hydroxytacrine maleate	3,05	282	Glyburide	1,32
83	Furafylline	2,51	183	Oxotremorine sesquifumarate	3,17	283	Minoxidil	3,12
84	nateglinide	2,06	184	Tacrine HCl	3,30	284	Tolazamide	2,10
85	Isoproterenol	3,09	185	Gallamine triethiodide	1,28	285	Nicorandil	0,12
86	AcetylcholineCl	4,47	186	Galanthamine HBr	2,27	286	Bexarotene	0,08
87	Atropine sulfate	2,26	187	Amiloride	2,46	287	Tranexamic acid	4,16
88	Decamethonium 2Br	2,53	188	Amlodipine	1,60	288	Celecoxib	1,71
89	Mecamylamine HCl	3,91	189	Diltiazem	1,45	289	Levetiracetam	3,84
90	Neostigmine Br	2,93	190	Nifedipine	1,89	290	Letrozole	2,29
91	Strychnine HCl	1,95	191	Nimodipine	1,56	291	Abamectin	0,75
92	Tubocurarine Cl (+)	1,07	192	Nitrendipine	1,81	292	Alfacalcidol	1,63
93	Butaclamol (+)	1,81	193	Verapamil	1,33	293	Anethole-trithione (anetholtrithion)	2,72
94	Apomorphine r (-)	2,44	194	Niguldipine HCl	1,01	294	Anastrozole	0,04
95	Chlorpromazine HCl	2,05	195	Flunarizine-2HCl	1,37	295	Bicalutamide	1,52
96	Domperidone	1,53	196	Gabapentin	3,82	296	Clodronate disodium	2,67
97	Fluphenazine 2HCl	1,49	197	Felodipine	1,70	297	Clindamycin palmitate	1,54
98	Pergolide mesylate	2,08	198	Cilnidipine	1,33	298	Vorinostat	0,10
99	Raclopride l-tartrate s(-)	1,88	199	Phenoxybenzamine HCl	1,92	299	Didanosine	2,77
100	Risperidone	1,59	200	Trifluoperazine 2HCl	1,36	300	Dolasetron	2,01

Nr.	Drug	final conc. [µM]
301	Enalaprilat	1,88
302	Fluvastatin Na	1,59
303	Fosinopril	1,16
304	Gemcitabine HCl	2,48
305	Ginkgolide a	0,06
306	Granisetron	2,09
307	Nedaplatin	2,16
308	Oxaliplatin	1,65
309	Anethole, trans-	4,41
310	Atazanavir	0,93
311	Mycophenolate mofetil	0,06
312	Etoricoxib	1,82
313	Clofarabine	2,15
314	Cabergoline	1,45
315	Dilazep	1,08
316	Ibandronate	2,05
317	Imipenem	2,18
318	Lomustine	2,80
319	Iloprost	1,81
320	Lomerizine HCl	1,39
321	Lofexidine	2,52
322	Meropenem	1,70
323	Nifekalant HCl	1,61
324	Octreotide	0,64
325	Oseltamivir	2,09
326	Pamidronic acid	2,78
327	Pramipexole	3,09
328	Triptorelin	0,50
329	Rifamycin sv	0,94
330	Risedronic acid	2,31
331	Rocuronium bromide	1,23
332	Ricobendazole	2,32
333	Sulbactam	2,80
334	Thiamphenicol glycinate	1,58
335	Tulobuterol	2,87
336	Vinorelbine	0,03
337	Vindesine	0,03
338	Salmeterol	1,57
339	Vincristine sulfate	0,03
340	Acemetacin	1,57
341	Acetylsalicylic acid	3,63
342	Acipimox	4,24
343	Aceclofenac	1,85
344	Acycloguanosine	2,90
345	3'-azido-3'-deoxythymidine	2,45
346	Allopurinol	4,80
347	Alendronate	2,63
348	Altretamine	3,11
349	Albendazole	0,10
350	Sumatriptan succinate	1,58
351	Amifostine	3,05
352	Dl-aminoglutethimide	2,81
353	4-aminosalicylic acid	4,27
354	5-aminosalicylic acid	4,27
355	Ampicillin trihydrate	1,87
356	Ampiroxicam	1,46
357	Apramycin	1,21
358	Artemisinin	2,31
359	Atenolol	2,45
360	Atracurium besylate	0,70
361	Azaperone	2,00
362	Vinblastine sulfate	0,03
363	Azithromycin	0,87
364	Aztreonam	1,50
365	Betamethasone	1,30
366	Bifonazole	2,11
367	Bisacodyl	1,81
368	Bromhexine HCl	1,74
369	Buspirone HCl	1,70
370	Canrenone	1,92
371	Carbadox	2,49
372	Carboplatin	1,94
373	Carbamazepine	2,77
374	Cefoperazone acid	1,01
375	Cefotaxime acid	1,43
376	Ceftazidime	1,20
377	Chloramphenicol	2,02
378	Chlormadinone acetate	1,46
379	Chlorambucil	2,15
380	Chlorpheniramine maleate	2,38
381	Chloroquine phosphate	2,04
382	Thalidomide	2,53
383	Ciprofloxacin	1,97
384	Citalopram	2,01
385	Clarithromycin	0,87
386	Climbazole	2,23
387	Clinafloxacin HCl	1,79
388	Clomiphene citrate	1,61
389	Amisulpride	1,77
390	Clopidogrel sulfate	2,03
391	Clobetasol propionate	1,40
392	Orphenadrine citrate	1,42
393	Corticosterone	1,89
394	Crotamiton	3,22
395	Cyclophosphamide monohydrate	2,50
396	Cyproterone acetate	1,57
397	Cyclocytidine HCl	0,12
398	Cytarabine	0,11
399	Dacarbazine	3,59
400	Danazol	1,94
401	Dehydroepiandrosterone	2,27
402	Desloratadine	2,10
403	Dextromethorphan HBr	2,41
404	Diclofenac, Na	2,21
405	2',3'-dideoxycytidine	3,09
406	Diethylstilbestrol	2,44
407	Diflunisal	2,61
408	Disulfiram	2,20
409	Doxazosin mesylate	1,45
410	Doxifluridine	2,65
411	Doxofylline	2,45
412	Doxycycline HCl	1,47
413	Enalapril	1,74
414	Enoxacin	2,04
415	Enrofloxacin	1,82
416	Ethisterone	2,09
417	Esomeprazole potassium	1,89
418	Estradiol	2,40
419	Estriol	2,27
420	Estrone	2,42
421	Etidronate 2Na	3,20
422	Etretinate	1,84
423	Trichloromethiazide	1,72
424	Famciclovir	2,03
425	Fenbendazole	0,09
426	Fenbufen	2,57
427	Fenoldopam mesylate	2,14
428	Fenoprofen	2,70
429	Fenofibrate	1,81
430	Finasteride	1,75
431	5-fluorouracil	5,02
432	Flurbiprofen	2,68
433	Fleroxacin	1,77
434	Amitriptyline HCl	2,08
435	Floxuridine	2,65
436	Fluocinolone acetonide	1,44
437	Flubendazole	0,08
438	Flutamide	2,37
439	Fluconazole	2,13
440	Formestane	2,16
441	Ftorafur	3,27
442	Furosemide	1,98
443	Ganciclovir	2,56
444	Gatifloxacin	1,74
445	Gentamycin sulfate	1,37
446	Gemfibrozil	2,61
447	Gliclazide	2,02
448	Glimepiride	1,33
449	Guaifenesin	3,30
450	Hexestrol	2,42
451	Hydrocortisone	1,80
452	Hydrocortisone 21-acetate	1,62
453	17 hydroxyprogesterone	1,98
454	Idoxuridine	1,85
455	Ifosfamide	2,50
456	Imiquimod	2,72
457	Indapamide	1,79
458	Itopride HCl	1,82
459	Itraconazole	0,93
460	Ketoprofen+D153	2,57
461	Levamisole HCl	3,20
462	Levonorgestrel	2,09
463	Levodopa	3,31
464	Levofloxacin HCl	1,81
465	Leflunomide	2,42
466	Lisinopril	1,61
467	Lomefloxacin HCl	1,86
468	Loratadine	1,71
469	Lorglumide	1,42
470	Losartan potassium	1,42
471	Mebendazol	0,09
472	Medroxyprogesterone-17 acetate	1,69
473	Mefenamic acid	2,71
474	Melphalan	0,09
475	Methyldopa	3,09
476	Methylprednisolone	1,75
477	Metoprolol tartrate	2,44
478	Methimazole	5,72
479	Metronidazole	3,82
480	Miconazole	1,57
481	Minocycline HCl	1,43
482	Mitoxantrone 2 HCl	0,06
483	Taxol	0,03
484	Myclobutanil	2,26
485	Nadifloxacin	1,81
486	Nabumetone	2,86
487	Naphazoline HCl	3,11
488	Nefazodone	1,39
489	Niflumic acid	2,32
490	Norethindrone	2,19
491	Norfloxacin	2,05
492	Nystatin	0,71
493	Ofloxacin	1,81
494	Oltipraz	2,89
495	Omeprazole	1,89
496	Oxcarbazepine	2,59
497	Oxiconazole nitrate	1,52
498	Oxacillin sodium monohydrate	1,63
499	Oxfendazole	2,07
500	Oxibendazole	0,10
501	Pantoprazole	1,70
502	Paroxetine HCl	1,98
503	Pazufloxacin	2,05
504	Pefloxacine mesylate	1,96
505	Penciclovir	2,58
506	Pentoxifylline	2,35
507	Penicillin v potassium	1,87
508	Phenylbutazone	2,12
509	Piperacillin	1,26
510	Pravastatin lactone	1,61
511	Pranoprofen	2,56
512	Prednisolone	1,81
513	Progesterone	2,08
514	Procarbazine HCl	2,95
515	Prothionamide	3,63
516	Prednisone	1,82
517	Pregnenolone	2,07
518	Primaquine phosphate	2,52
519	Praziquantel	2,09
520	Pyrantel pamoate	1,10
521	Quinacrine 2HCl dihydrate	0,07
522	Quinapril HCl	1,49
523	Racecadotril	1,70
524	Ranolazine 2HCl	1,53
525	Ramipril	1,57
526	Rebamipide	1,76
527	Ribavirin	2,68
528	Nelfinavir mesylate	0,98
529	Rimantadine HCl	3,65
530	Propranolol	2,52
531	Roxatidine acetate HCl	1,88
532	Roxithromycin	0,78
533	Rufloxacin	1,80
534	Sarafloxacin HCl	1,70
535	Scopolamine n-butylbromide	1,81
536	Scopolamine HBr	2,15
537	Secnidazole	3,53
538	Sibutramine HCl	2,34
539	Sparfloxacin	1,67
540	Spironolactone	1,57
541	Stanozolol	1,99
542	Streptomycin sulfate	0,04
543	Sulfadoxine	2,11
544	Sulfadiazine	2,61
545	Sulfadimethoxine	2,11
546	Sulfasalazine	1,64
547	Tamsulosin HCl	1,60
548	Telmisartan	1,27
549	Tenoxicam	1,94
550	Terazosin HCl	1,69
551	Tetracycline	1,47
552	Tenatoprazole	1,89
553	Temozolomide	3,37
554	Tibolone	2,09
555	Tioconazole	1,69
556	Tinidazole	2,64
557	Tobramycin (free base)	1,40
558	Tosufloxacin	1,62
559	Topotecan	0,06
560	Toremifene	1,61
561	Tolfenamic acid	2,50
562	Tolmetin Na	0,10
563	Amoxicillin	1,79
564	Tramadol HCl	0,10
565	Triamcinolone	1,66
566	Trimethoprim	2,25
567	Tropisetron HCl	0,09
568	Tylosin tartrate	0,03
569	Valaciclovir	2,02
570	Vecuronium Br	0,05
571	Venlafaxine HCl	2,36
572	Vidarabine	0,02
573	Iproniazid	3,65
574	Benzamil HCl	1,83
575	Bupivacaine HCl	2,27
576	Astemizole	0,06
577	Ketotifen fumarate	2,11
578	Levocabastine HCl	0,03
579	Naloxone HCl	2,00
580	Naloxonazine 2HCl	0,04
581	Harmine	3,08
582	Cinanserin	1,92
583	Dibenzepine HCl	2,21
584	Dihydroergocristine mesylate	1,07
585	Fluperlapine	2,11
586	Fluoxetine HCl	2,11
587	Ondansetron	0,04
588	Ketanserin tartrate	1,65
589	Tiotropium Br	1,38
590	Mesoridazine besylate	0,07
591	Thioridazine HCl	1,76
592	Cilostamide	0,08
593	Etazolate	0,09
594	Amrinone	0,14
595	Irsogladine maleate	2,55
596	Milrinone	0,12
597	Rolipram	2,37
598	Siguazodan	0,09
599	Trequinsin HCl	1,48
600	Vinpocetine	1,86

Nr.	Drug	final conc. [µM]	
601	Zaprinast	2,41	
602	Zardaverine	0,10	
603	Alprostadil	1,84	
604	Misoprostol	0,07	
605	Artesunate	1,70	
606	Picotamide	1,74	
607	Butoconazole nitrate	1,38	
608	Mifepristone	0,03	
609	Megestrol acetate	1,70	
610	Melengestrol acetate	0,07	
611	Tamoxifen citrate	0,07	
612	Amprenavir	1,29	
613	Aprepitant	1,22	
614	Bosentan	1,18	
615	Efavirenz	2,07	
616	Taurocholic acid, sodium salt hydrate	1,27	
617	Miglustat	2,98	
618	Fulvestrant	1,08	
619	Methysergide	0,02	
620	Esmolol	2,21	
621	Pantothenic acid	2,98	
622	Capecitabine	1,82	
623	Phenylpropanolamine	4,32	
624	Succinylcholine	2,25	
625	L-thyroxine[(3-[4-(4-hydroxy-3,5-diiodophenoxy)-3,5-diiodophenyl]-l-alanine]	0,84	
626	Cyproheptadine	2,27	
627	Benzydamine	2,11	
